# Differential expression of antioxidant system genes in honey bee (*Apis mellifera* L.) caste development mitigates ROS-mediated oxidative damage in queen larvae

**DOI:** 10.1590/1678-4685-GMB-2020-0173

**Published:** 2020-11-13

**Authors:** Douglas Elias Santos, Anderson de Oliveira Souza, Gustavo Jacomini Tibério, Luciane Carla Alberici, Klaus Hartfelder

**Affiliations:** 1 Universidade de São Paulo, Faculdade de Medicina de Ribeirão Preto, Departamento de Biologia Celular e Molecular e Bioagentes Patogênicos, Ribeirão Preto, SP, Brazil.; 2 Universidade de São Paulo, Faculdade de Ciências Farmacêuticas de Ribeirão Preto, Departamento de Ciências BioMoleculares, Ribeirão Preto, SP, Brazil.

**Keywords:** Social insect, oxidative damage, reactive oxygen species, antioxidant system, Apis mellifera

## Abstract

The expression of morphological differences between the castes of social bees is triggered by dietary regimes that differentially activate nutrient-sensing pathways and the endocrine system, resulting in differential gene expression during larval development. In the honey bee, *Apis mellifera*, mitochondrial activity in the larval fat body has been postulated as a link that integrates nutrient-sensing via hypoxia signaling. To understand regulatory mechanisms in this link, we measured reactive oxygen species (ROS) levels, oxidative damage to proteins, the cellular redox environment, and the expression of genes encoding antioxidant factors in the fat body of queen and worker larvae. Despite higher mean H_2_O_2_ levels in queens, there were no differences in ROS-mediated protein carboxylation levels between the two castes. This can be explained by their higher expression of antioxidant genes (*MnSOD*, *CuZnSOD, catalase,* and *Gst1*) and the lower ratio between reduced and oxidized glutathione (GSH/GSSG). In worker larvae, the GSG/GSSH ratio is elevated and antioxidant gene expression is delayed. Hence, the higher ROS production resulting from the higher respiratory metabolism in queen larvae is effectively counterbalanced by the up-regulation of antioxidant genes, avoiding oxidative damage. In contrast, the delay in antioxidant gene expression in worker larvae may explain their endogenous hypoxia response.

## Introduction

The evolution of morphologically and functionally different adult castes represents a major transition in the highly eusocial organization of insects and vertebrates, as it literally sets a point of no return back to a communal or solitary lifestyle (Wilson and Hölldobler, 2005). It is also a major disruption in the reproduction *vs.* longevity trade-off paradigm. In social Hymenoptera (ants, bees and wasps), this divergence is restricted to the female sex, with highly fertile, long-lived queens in contrast with subfertile to sterile, short-lived workers. In terms of developmental regulation, this divergence is generally triggered by environmental factors, especially the type of diet provided to the larvae, and only exceptionally is it based on genotype differences (Schwander *et al.*, 2010).

In the honey bee, *Apis mellifera*, a young female larva has the potential to develop into either a queen or a worker, contingent on the nutritional regime to which it is exposed during larval development (Haydak, 1970; Hartfelder *et al.*, 2015), and as a consequence of these distinct feeding regimes, adult queens and workers differ not only in their external and internal morphologies and their reproductive capacities, but also in their lifespans. Hence, understanding this remarkable developmental plasticity requires insights into how the differential larval feeding regimes are converted into meaningful systemic responses. One of the primary responses is the differential activation of the endocrine system, primarily of the *corpora allata* that produce and release juvenile hormone (JH) at much higher rates in queen than in worker larvae (Rachinsky and Hartfelder, 1990). The resulting elevated JH levels in the hemolymph of queen larvae (Rembold, 1987) prevent the onset of programmed cell death in the larval ovaries and, thus, guarantee the high reproductive capacity of the adult queens (Hartfelder *et al.*, 2018).

Further insights into systemic responses emerged once the honey bee genome had been sequenced. These included nutrient sensing pathways, such as insulin/insulin-like (IIS) and TOR signaling, both predicted to be up-regulated in queen larvae (Wheeler *et al.*, 2006; Patel *et al.*, 2007). Strikingly though, during the major growth phase in the fourth and fifth larval instars, queen larvae exhibited a drastic reduction in the expression levels of the two insulin receptor genes (de Azevedo and Hartfelder, 2008), and equally surprising was the finding that the transcript levels of the *tor* gene are actually higher in worker larvae than in queens (Hartfelder *et al.*, 2015). Signaling via the EGF receptor pathway, which had been proposed as a key response to the feeding of queen larvae with royalactin protein (Kamakura, 2011), also showed inconsistencies, as the honey bee *egfr* gene was found higher expressed in worker and not in queen larvae (Hartfelder *et al.*, 2015). Furthermore, the function of royalactin protein, which had been proposed to be the queen-determining factor (Kamakura, 2011), is rather controversial (Buttstedt *et al.*, 2016).

Such seemingly unorthodox patterns in highly conserved eukaryotic growth control pathways made us look for alternative, integrative signaling modules. One of these, hypoxia signaling, had previously been shown to directly communicate with the IIS/TOR pathway in *Drosophila melanogaster* (Dekanty *et al.*, 2005). Strikingly, when investigating the expression levels of the three hypoxia core genes in honey bee larvae, we found that all of these, and especially so the HIF-1α homolog *Amsima*, are up-regulated in worker development (Azevedo *et al.*, 2011). This was interpreted as an endogenous hypoxia response, as there is no evidence that queen and worker larvae, which are reared next to each other in the hive, would be exposed to different environmental oxygen conditions.

A crucial factor in the hypoxia response is the stabilization of HIF-1α protein through inhibition of the enzymatic activity of an oxygen-sensing prolyl hydroxylase (PHD). The inhibition of PHD function and consequent stabilization of HIF-1α has been associated with mitochondria-derived reactive oxygen species (ROS), such as hydrogen peroxide (Li *et al.*, 2014; Schieber and Chandel, 2014). Superoxide radicals (O_2_^·-^) are mainly produced as a by-product of aerobic respiration during electron transfer reactions in the respiratory chain, or via the activity of NADPH oxidases (Lambeth, 2004; Kowaltowski *et al.*, 2009).

For years, ROS were considered as the villains of aging-related degenerative processes of cell function, as they promote oxidative stress that causes damage to lipids, proteins, and DNA (Cross *et al.*, 1987). Cells are capable of minimizing such damaging ROS effects by making use of a variety of antioxidant systems. The first line of defense is mainly represented by enzymes, such as the mitochondrial and cytoplasmatic superoxide dismutases (MnSOD and CuZnSOD, respectively) that detoxify O_2_^·-^ into the less reactive hydrogen peroxide (H_2_O_2_). The second line then includes catalase, glutathione peroxidase, and the reduced glutathione tripeptide (GSH) (Lei *et al.*, 2016). Although ROS do induce oxidative stress, especially when at high concentrations, it is now commonly accepted that ROS are also vitally important signaling factors that allow cellular and systemic adaptations to changes in the oxidative environment and to nutrient availability animals (Rhee, 2006; Amigo *et al.*, 2016; Pelster *et al.*, 2020; Sies and Jones, 2020), including insects (Lennicke and Cochemé, 2020).

The adaptive response between a cell's or organism's need to produce variable levels of ATP depending on energy requirement and the balance in ROS levels has been termed mitochondrial hormesis or mitohormesis (Hood *et al.*, 2018). Since the level of ATP production in a given cell is directly related to mitochondrial number by fission of fusion (Westermann, 2010), as well as their intracellular distribution and density of their inner cristae (Strohm and Daniels, 2003) ROS production will necessary be linked to such changes in mitochondrial dynamics (Heine and Hood, 2020). Hence, the expression levels of antioxidant factors and enzymes will be of critical importance.

In our previous work (Santos *et al.*, 2016) we aimed to understand the above-mentioned endogenous hypoxia response in honey bee larvae from a cellular and biochemical perspective. Specifically, we investigated the mitochondrial activity by means of high resolution respirometry, mitochondrial number, and their intracellular distribution and ultrastructure in the larval fat body, which is the metabolic center of insects (Arrese and Soulage, 2010). We could show that honey bee queen larvae have a considerably higher mitochondrial respiratory activity, that they have more mitochondria, and a unique, cup-shaped mitochondrial conformation. This led us to ask whether and how queen and worker larvae may adjust their ROS levels, which we expected to be higher due to the queens’ higher respiration rates, to avoid oxidative damage, and how this integrates with hypoxia signaling in the caste differentiation process.

Here, we measured H_2_O_2_ levels, protein carboxylation, the levels of reduced and oxidized glutathione, and quantified the transcript levels of genes encoding candidate antioxidant enzymes in the fat body of queen and worker larvae. We found that although queen larvae have higher ROS levels, their higher transcript levels for antioxidant system genes can apparently balance the cellular redox state and, thus, mitigate oxidative damage.

## Material and Methods

### Honey bee larvae

Fat body tissue was dissected from queen and worker larvae reared according to standard apicultural practice in hives of Africanized hybrid honey bees (*A. mellifera* L) kept in the experimental apiary of the Department of Genetics, University of São Paulo, Ribeirão Preto, Brazil. For the assays we used fourth-instar larvae (L4) and the following substages of the fifth larval (L5) instar: F1, F2 and F3, which are the stages when larvae are still fed by nurse bees, and the first substage of the cocoon-spinning phase, S1, when the larvae stopped feeding and start to prepare for metamorphosis. These stages were chosen because caste fate is still relatively flexible in the fourth instar, and then becomes gradually fixed in the feeding stages of the fifth instar (Dedej *et al.*, 1998; Leimar *et al.*, 2012).

### Fluorometric quantification of H_2_O_2_ levels

The fat body tissue was dissected from for each of the developmental stages of queen and worker larvae, fragmented into small pieces, and these were loaded into a reaction cuvette containing 2 mL of MiR05 respiration buffer (20 mM HEPES, 10 mM KH_2_PO_4_, 110 mM sucrose, 20 mM taurine, 60 mM K-lactobionate, 0.5 mM EGTA, 3 mM MgCl_2_, 1 g/L fatty acid-free BSA, pH 7.1). H_2_O_2_ production was measured at 37 °C following the addition of 4 μL of Horseradish Peroxidase (1000 U/mL) and 2 μL of 50 μM Amplex® Red Hydrogen Peroxide/Peroxidase Assay Kit (Molecular Probes/ThermoFisher, Eugene, USA). The reaction was monitored for 10 min in a fluorescence spectrophotometer (Model F-4500, Hitachi, Tokyo, Japan) using 563/587 nm excitation/emission filters. The reaction occurred under constant stirring to facilitate the diffusion of the H_2_O_2_ produced by the fat body cells into the reaction medium. After curve stabilization, 40 nM of pyruvate was added. The raw data were normalized by the total amount of protein of the samples measured by the Bradford method. A standard curve was established by sequential addition of hydrogen peroxide (10 nM) to the reaction medium. The assays were run in triplicate for each developmental stage of the two castes.

### Quantification of protein carbonylation and reduced (GSH) and oxidized (GSSG) glutathione levels

Protein carbonylation and reduced (GSH) and oxidized (GSSG) glutathione levels were assessed in fat body homogenates from five independent biological samples of each of the developmental stages of queen and worker larvae. The samples were homogenized in cold 0.1 M Tris-HCl buffer (pH 7.4), centrifuged at 1500 × *g* for 10 min at 4 °C, and the supernatant containing water-soluble proteins was collected.

The presence and quantity of protein carbonyl groups was assessed through the selective binding of 2,4-dinitrophenyl hydrazine (DNPH) to protein carbonyl groups (Reznick and Packer, 1994). Measurements were taken in a Cary 50MPR spectrophotometer (Varian Ltd., Melbourne, Australia) at 340 nm. The assays were run in quintuplicate for each developmental stage and caste.

GSH and GSSG levels were measured for the same samples by the fluorimetric ortho-phthalaldehyde method (Hissin and Hilf, 1976) using a 350/420 nm excitation/emission filter combination in a Synergy 2 spectrophotometer (BioTek Instruments, Winooski, USA). The raw data were normalized by the total protein concentration of each sample. The assays were run in quintuplicate for each developmental stage and caste.

### Fold change of candidate gene transcript levels

The expression of antioxidant-encoding genes directly involved in the degradation and neutralization of superoxide radicals and H_2_O_2_ were quantified by real-time PCR. The primer sequences published by Corona *et al.* (2005) were used for transcript level quantification of the manganese-dependent superoxide dismutase (*MnSOD*), cytoplasmatic copper and zinc-dependent superoxide dismutase 1 (*CuZnSOD*), glutathione peroxidase *(Gtpx-1)*, catalase *(Cat),* and glutathione S-transferase-1 (*Gst-1*) genes.

RNA was extracted using TRIzol (Life Technologies, Carlsbad, CA, USA) from fat body samples of each developmental stage and caste. Residual DNA was removed by treatment with RNase-free DNase (Life Technologies). First strand cDNA was synthesized by reverse transcription using the Superscript II enzyme and Oligo(dT)_12-18_ primer (both from Life Technologies, Carlsbad, CA, USA).

Quantitative RT-PCR (RT-qPCR) analyses were set up using 1.0 μL of cDNA (diluted 1:10), 5 μL of Power SYBR^®^PCR Green Master Mix (Life Technologies), 0.5 μL of each forward and reverse primer (10 pmol/μL) and 3 μL of deiononized water (MilliQ, Millipore, Billerica, MA, USA), completing a final volume of 10 μL. Reactions were run in a Real-Time PCR StepOne Plus system (Life Technologies) system under the following conditions: 50 °C for 2 min, 95 °C for 10 min, 40 cycles of 95 °C for 15 s and 60 °C for 1 min, followed by melting curve analysis (95 °C for 15 s, 60 °C for 1 min and 95 °C for 15 s).

For each biological sample (n=3 per developmental stage and caste), technical triplicates were run. Relative transcript levels were calculated using the 2^–DDCt^ method (Livak and Schmittgen, 2001). The honey bee *rpl32* gene (formerly known as *rp49*), which has been validated for honey bee RT-qPCR assays (Lourenço *et al.*, 2008), served as endogenous control gene, and one of the fourth-instar worker samples was used as calibrator for each gene.

### Statistical analyses

After checking conformity with the assumptions for parametric testing, the data were analyzed statistically by two-way ANOVA and post-hoc Bonferroni *t-*tests using caste and developmental stage as factors. Levels of *p*<0.05 were considered statistically significant. GraphPad ©Prism 5.01 software (San Diego, CA, USA) was used for the statistical tests and also to prepare the graphs.

## Results

### ROS (H_2_O_2_) levels in larval fat body

**Figure 1:**
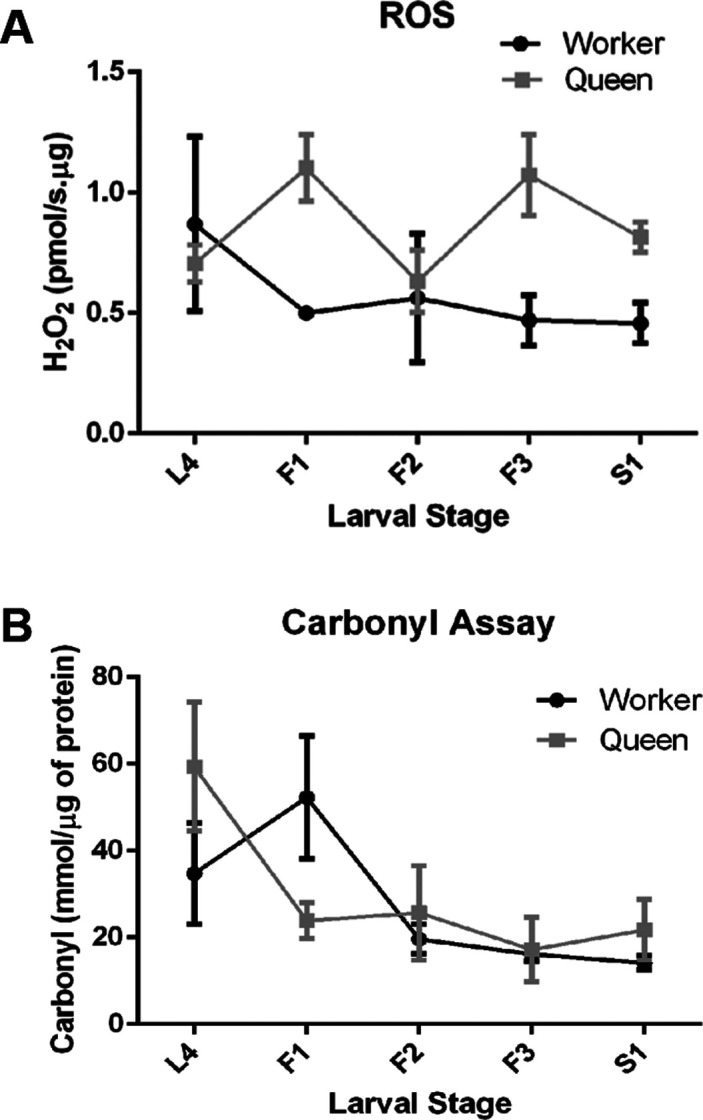
ROS levels and oxidative damage in fat body cells of honey bee queen and worker larvae. (A) H2O2 production rates were measured by the fluorometric Amplex® Red Hydrogen Peroxide/Peroxidase Assay in freshly dissected fat body tissue of the fourth (L4) and the first four substages of the fifth larval instar (F1-S1). Shown are means ± SEM (n= 3). (B) ROS-mediated damage to proteins was evaluated spectrophotometrically by the selective binding of 2,4-dinitrophenyl hydrazine (DNPH) to protein carbonyl groups in fat body homogenates of queen and worker larvae. Shown are means ± SEM (n=5). For statistical analysis results see text.

ROS production measured by means of the fluorometric Amplex® Red Hydrogen Peroxide/Peroxidase Assay revealed significantly higher mean levels in queen larvae across the developmental period in question (Figure 1A; two way ANOVA, caste factor F_(1, 21)_ = 7.656, *p*=0.0117). No significant differences were reported for developmental stage, or interaction between caste and developmental stage.

### ROS-related protein carbonylation and the cellular redox state in larval fat body

To assess oxidative stress parameters we first measured protein carbonylation levels As shown in Figure 1B, protein carbonylation levels progressively declined as the larvae gradually approached the entry to metamorphosis (two way ANOVA, factor developmental stage, F_(4, 39)_ = 4.356, *p*=0.0052). There were no significant differences for the factor caste or the interaction term between developmental time and caste.

**Figure 2:**
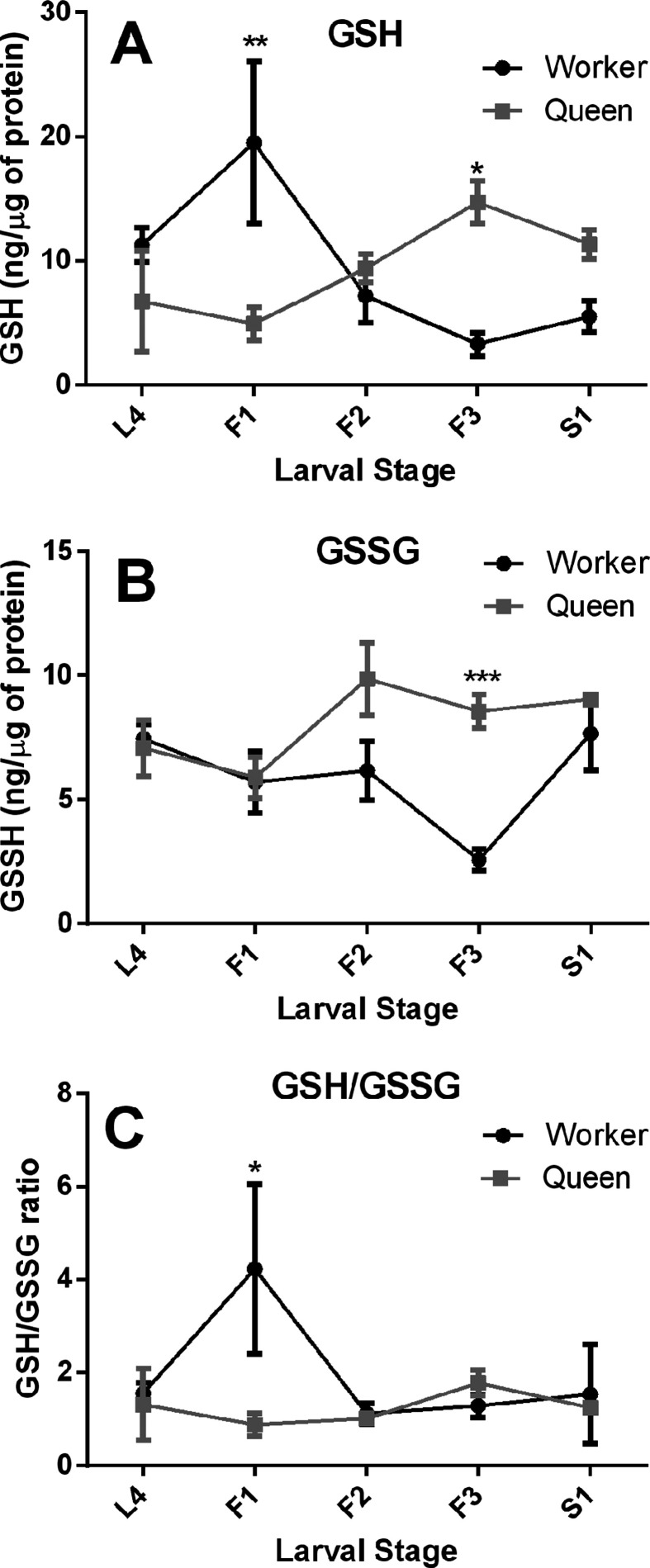
Redox state of fat body cells in honey bee queen and worker larvae. (A) reduced (GSH) and (B) oxidized (GSSG) glutathione levels; (C) GSH/GSSG ratio. GSH and GSSG levels were measured separately by the fluorimetric ortho-phthalaldehyde method and normalized to total protein. The larval stages are the same as those shown in Figure 1. Shown are means ± SEM (n=5). Statistical differences between the two castes were tested by two-away ANOVA with Bonferroni post-hoc tests (*p<0.05, **p<0.01, ***p<0.001).

Next, we measured the levels of reduced (GSH) and oxidized glutathione levels (GSSG) and calculated their ratio, which represents the redox state in the larval fat body tissue. The levels of GSH, GSSG, and the GSH/GSSG ratio are important indicators of the redox state of the cellular environment and, consequently, of the oxidative stress level (Schafer and Buettner, 2001). With respect to their GSH levels (Figure 2A), the two castes showed a directly inverted time course, with high initial levels in workers, reflected in the fact that the interaction factor in the two-way ANOVA was statistically highly significant (F_(4, 39)_ = 6.51, *p*<0.005), but not the caste or developmental stage factors. However, the Bonferroni post-hoc tests revealed significantly higher GSH levels for F1-stage workers (*t*=3.412, *p*<0.01) and significantly higher levels for F3-stage queens (*t*=3.22, *p*<0.05). In contrast, for the GSSG levels (Figure 2B) we found statistical differences for all three terms (two way ANOVA, caste, F_(1, 39)_ = 11.14, *p*=0.0019; developmental stage, F_(4, 39)_ = 3.098, *p*=0.0263; interaction F_(4, 39)_ = 3.27, *p*=0.021). For the GSH/GSSG ratio (Figure 2C) there were no overall significant differences for the factors caste, developmental stage, or interaction, but the Bonferroni post-hoc test showed a significant difference in the redox state of fat body cells for the early fifth instar larvae (F1-stage, *t*=2.936, *p*<0.05). This is primarily due to a transient GSH peak seen in this same stage.

### Transcript levels of antioxidant genes in the fat body of honey bee larvae

By measuring the transcript levels of two honey bee genes encoding enzymes that are critically involved in the conversion of intracellular oxygen radicals to hydrogen peroxide (MnSOD, CuZnSOD), as well as genes encoding catalase, glutathione peroxidase (Gtpx-1), and reduced glutathione protein (Gst-1), we intended to obtain insights into regulatory mechanisms related to the observed fluctuation in ROS levels and the cellular redox state inferred from the GSH/GSSG ratio.

**Figure 3:**
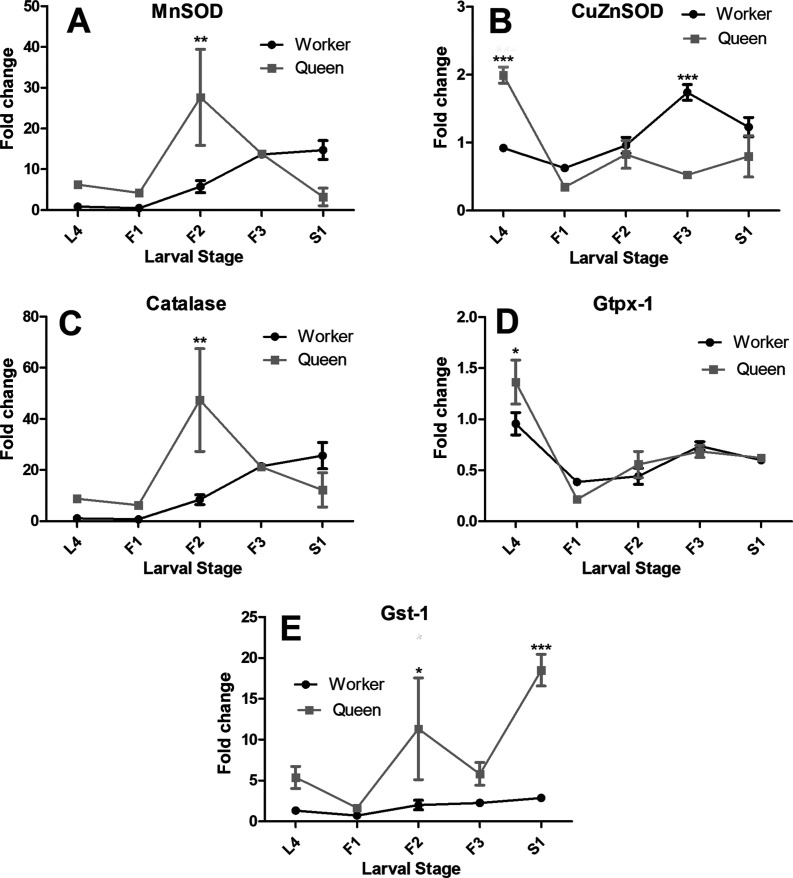
Antioxidant system genes fold change in the fat body of honey bee queen and worker larvae. Relative transcript levels were quantified by RT-qPCR for: (A) MnSOD, (B) CuZnSOD, (C) catalase, (D) Gtpx-1 and (E) Gst1. Shown are the means ± SEM of three biological samples per stage and caste, each analyzed in triplicate. Fold change was calculated by the 2–DDCt method (Livak and Schmittgen, 2001) using rpl32 expression for normalization and one of the fourth-instar worker samples as calibrator for each gene. The larval stages are the same as those shown in Figure 1. Statistical analysis was performed by two-way ANOVA and Bonferroni post-hoc tests (*p<0.05, **p<0.01, ***p<0.001).

The transcript levels for the two genes encoding superoxide dismutases showed statistically significant differences for the two castes (Figure 3A,B). For instance, *MnSOD* expression exhibited a marked peak in the F2 stage of queen larvae (two-way ANOVA, factor caste, F_(1,20)_ = 5.063, *p*=0.0055; Bonferroni post-hoc test *t*=3.942, *p*<0.01). The transcript levels for *CuZnSOD* showed significant differences, both with respect to caste (F_(1,20)_ = 12.34, *p*<0.001) and developmental stage (F_(1,20)_ = 4.766, *p*=0.0411). *MnSOD* expression was significantly up-regulated in fourth instar queen larvae when compared to workers (Bonferroni post-hoc test *t*=5.309, *p*<0.001), but later, at the end of the larval feeding stage in the fifth instar (F3), it was higher expressed in the worker caste (Bonferroni post-hoc test *t*=5.999, *p*<0.001).

The expression levels of *catalase* (Figure 3C) followed the general pattern seen for *MnSOD*, with statistically significant differences for the factor caste (F_(1,20)_ = 4.718, *p*=0.0076), with a clear expression peak in the F2 stage of queens (Bonferroni post-hoc test *t*=3.967, *p*<0.01). For *Gtpx-1* (Figure 3D) the caste difference was also significant (F_(1,20)_ = 23.05, *p<*=0.0001), due to a caste difference in transcript levels in fourth instar larvae (Bonferroni post-hoc test *t*=3.057, *p*<0.05). The *Gst-1* transcript levels (Figure 3E) differed significantly, both with respect to caste (F_(1, 20)_ = 5.662, *p*=0.032) and developmental stage (F_(4, 20)_ = 23.85, *p*<0.0001), the main differences residing in the up-regulated in the *Gst-1* expression in F2- and S1-stage queen larvae (Bonferroni post-hoc test for F2 *t*=3.046, *p*<0.05; for S1 *t*=5.099, *p*<0.001).

When changing the perspective and looking at the temporal expression profiles for all the five genes together, the two castes showed clearly distinct overall patterns concerning antioxidant gene expression (Figure S1). While in queen larvae, a strong expression of antioxidant genes was apparent in the F2 stage of the fifth instar, worker larvae exhibited a gradual increase in antioxidant gene expression as they finished the feeding stage (F3) and became spinning-stage larvae (S1). Interestingly though, in both cases the respective caste-specific expression patterns were dominated by the *catalase* and *MnSOD* transcript levels.

## Discussion

This study has two main findings, first, the elevated ROS levels in the fat body of queen larvae (Figure 1A), that correlate with the previously found higher mitochondrial activity in queen larvae (Eder *et al.*, 1983; Santos *et al.*, 2016), and second, a major difference in the time course of antioxidant system gene expression. Their up-regulated expression in early fifth-instar queen larvae (Figure 3 and Figure S1) apparently counterbalances the potentially negative effects of the higher respiratory metabolism of queen larvae and, thus, may mitigate a potential ROS-related damage to proteins (Figure 1B). The coordinated, transient overexpression of antioxidant genes in early fifth instar larvae may also be relevant for controlling oxidative stress, as expressed in the well balanced GSH/GSSG ratio throughout queen development (Figure 2). In contrast, worker larvae exhibited a marked peak in their GSH/GSSG ratio early in the fifth instar, primarily associated with their high GSH levels in this stage, and we hypothesize that this unbalanced redox state may be due to the delay seen in antioxidant gene expression.

Overall, it is interesting to note that both castes appear to primarily invest in the expression of two genes of the antioxidant defense-system response to regulate ROS levels in their fat body, which is the major metabolic organ of insects. These are the genes encoding an intramitochondrial manganese-dependent superoxide dismutase (MnSOD) and catalase (Figures 3A and S1). The fact that the intramitochondrial MnSOD gene is much higher expressed than its cytoplasmatic counterpart CuZnSOD, indicates that most of the ROS present in the larval fat body is produced within the mitochondria, as a result of electron transport chain activity.

The observation that the overall timing of antioxidant gene expression is delayed in workers until the late feeding stage in the fifth instar is also of interest, as this is possibly associated with their lower capacity of ATP production at higher physiological demand in comparison to queens (Santos *et al.*, 2016). In contrast, in queen larvae, *MnSOD* and *catalase* are strongly expressed in the middle of the last-instar feeding stage, the F2-stage. This is in agreement with the mitochondrial dynamics seen in the fat body of honey bee larvae, where a marked increase in mitochondrial number was detected in queens at the transition from the F2 to the F3 stage (Santos *et al.*, 2016).

ROS are Janus-face molecules; they can be both vital signaling molecules, as well as cause damage to biomolecules (Schieber and Chandel, 2014). At low intracellular concentrations they function as a redox signaling system through which physiological processes can be adaptively adjusted to environmental conditions (Hsieh and Hsu, 2013). In redox signaling, nanomolar ROS concentrations cause the oxidation of thiol residues of cysteins in proteins, and this oxidation can be reverted by glutaredoxins or thioredoxins (Winterbourn and Hampton, 2008). Such reversible thiol group oxidation may occur in transcription factors or proteins involved in intracellular signal transduction, and this will change and adjust cell growth and proliferation rates. In contrast, at higher intracellular ROS levels, such thiol residues are further oxidized, and the conformation state of proteins becomes irreversibly altered, reflecting oxidative damage.

The Janus-face function of ROS poses the question as to which of the two ROS functions is the prevalent one in the context of honey bee caste development. For queen larvae, their well-balanced redox state, even at elevated ROS levels, and the dynamic overexpression of antioxidant genes indicates that in queen larvae, ROS may be primarily functioning as signaling factors. In contrast, the redox state of fat body cells of worker larvae is unbalanced at the beginning of the fifth instar, and there is a delay in the expression of important antioxidant system genes. In F1-stage worker larvae, the timing of the redox state change was found to coincide with an increase in mean protein carbonylation levels (Figure 1B) and high GSH (Figure 2A), despite a low ROS level (Figure 1A). So, the question is, are worker larvae so to speak “living on the edge” in their mitochondrial hormetic state, tending more towards oxidative stress than towards redox signaling?

The distinct feeding regimes that queen and worker larvae experience during postembryonic development are the key factors for their divergent developmental trajectories, and these feeding regimes, especially with respect to sugar content in the larval diets, have been incorporated into a mathematical model for honey bee development (Leimar *et al.*, 2012). The data obtained here on ROS and antioxidant system gene expression in larvae now raise the question as to the presence of compounds with potential antioxidant properties in these diets, and indeed, analyses of royal jelly have indicated biological activity against lipid peroxidation and reduction of the superoxide radical (Liu *et al.*, 2008). However, the respective compounds have not yet been fully identified, and nor is it known whether such activity may also be present in worker jelly, which is produced and secreted by the same glands in the head of nursing workers.

Evidence for a strong connection between diet and the metabolic state of queen and worker larvae has long been reported from differential gene expression studies (Corona *et al.*, 1999; Evans and Wheeler, 2001). Similarly, the analysis of queen and worker larval methylomes (Foret *et al.*, 2012) revealed several highly conserved genes encoding nutrient sensing and metabolic factors as enriched among the differentially methylated genes. A recent epigenomics analysis integrating histone posttranslational modifications, DNA methylation and transcriptome data for larval honey bee brains (Wojciechowski, 2018) also presented strong evidence for an epigenetic up-regulation of physio-metabolic genes in queen larvae in comparison to worker larvae. The mechanistic connection of the nutrient-sensing pathways with the epigenetic marks and differential gene expression patterns, however, is still a black box. Our previous findings indicated that the endogenous hypoxia response in honey bee worker larvae (Azevedo *et al.*, 2011) may be explained by their lower mitochondria number (Santos *et al.*, 2016) and the unbalanced cellular redox state shown here for their main metabolic tissue, the larval fat body. This would point towards redox signaling and mitochondrial dynamics as a mechanistic link between nutrient sensing and differential gene expression in honey bee caste development.

**Figure 4:**
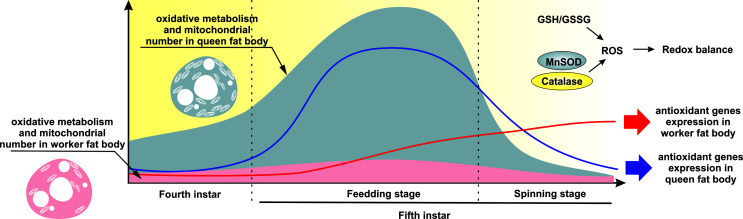
Summary graphical representation and hypothesis based on the obtained results. The high mitochondrial activity in queen and the much lower activity in worker larvae during the last two larval instars (Santos et al., 2016) leads to higher ROS production in queens. The potential oxidative damage of these higher ROS levels in queens is effectively balanced by an expression peak of antioxidant system genes, especially MnSOD and catalase and a balanced cellular redox environment (GSH/GSSG). In worker larvae, despite lower ROS levels, the cellular redox environment is unbalanced and antioxidant gene expression is delayed. These factors likely contribute to the overexpression of hypoxia signaling genes in worker (Azevedo et al., 2011).

Alterations in the cellular redox environment are known to lead to HIF-1α stabilization through the inhibition of prolyl hydroxylase activity in mammalian cells (Li *et al.*, 2014), and we have previously shown that the honey bee homologs of HIFα and HIFβ are up-regulated in worker larvae at the transition from the fourth to the fifth larval instar (Azevedo *et al.*, 2011), but so is the expression of the insect PHD homolog *fatiga*, the negative regulator of hypoxia signaling. Though apparently a contradiction, a study on HIF-dependent and HIF-independent transcriptional responses to hypoxia in *Drosophila* postembryonic development has revealed that *fatiga* expression can in fact also be induced by hypoxia (Li *et al.*, 2013). Furthermore, this same study showed that the estrogen-related receptor (ERR) is an interaction partner of HIFα in this context. ERR is an important regulator of carbohydrate metabolism (Tennessen *et al.*, 2011) and, interestingly, expression of the honey bee homolog of ERR is strongly up-regulated in queen larvae during the fifth instar feeding phase (Santos *et al.*, 2016). This indicates a very intricate balance in the connectivity between these phylogenetically ancient regulators of cell physiology, as well as their relationship with the complex processes underlying the nutrition-driven phenotypic plasticity in the honey bee. In Figure 4 we synthetically represent these findings and their integration.

With this in mind it is actually interesting to speculate as to whether and how the metabolic settings in the postembryonic stages of development may actually also be of relevance in the adult honey bee castes. The transition in metabolic settings from the larval to the adult stage is, however, not trivial, because the larval fat body of holometabolous insects is completely lysed during metamorphosis, and becomes rebuilt from a yet unknown population of stem cells of mesodermal origin for adipocytes and urocytes, and of ectodermal origin for oenocytes (Arrese and Soulage, 2010). Nonetheless, in a pioneering study, Wang *et al.* (2016) showed that workers that had been starved as larvae for only a few hours showed an improved metabolic response and better survival after subsequent adult starvation. Furthermore, in the context of aging and immunosenescence, studies have flourished in recent year on the mitochondrial energy metabolism, ROS, and antioxidant factors in adult honey bee queens and workers (Chuang and Hsu, 2013; Hsieh and Hsu, 2013; Hsu and Chuang, 2014; Hsu and Lu, 2015), and in workers performing different age-related tasks (Cervoni *et al.*, 2017; Margotta *et al.*, 2018). The differences in oxidative metabolism found in these studies indicate that also in adult queens and workers there is a very fine-tuned balance between redox signaling and oxidative stress. In a broader sense, these results on how mitochondrial activity and ROS functions can be related to and affect the expression of metabolic and antioxidant genes throughout the life cycle of this highly eusocial insect may provide a novel perspective for insights concerning the major shift in the reproduction/longevity trade-off paradigm that occurred in the evolution of highly eusocial insects and vertebrates (Flatt *et al.*, 2013; Flatt and Partridge, 2018), where queens are both exceptionally long-lived and reproductive, while workers are short-lived and subfertile or even sterile.

## Supplementary material

The following online material is available for this article:

Figure S1 Fold change of antioxidant system genes from a caste perspective.
