# Supplementary material for: Differential expression of antioxidant system genes in honey bee (Apis mellifera L.) caste development mitigates ROS-mediated oxidative damage in queen larvae
Source: Genet Mol Biol. 2020 Nov 13;43(4):e20200173. doi: 10.1590/1678-4685-GMB-2020-0173 (PMC7783730; doi:10.1590/1678-4685-GMB-2020-0173)
Supplement: Supplementary file 1 [file 1415-4757-GMB-43-4-e20200173-s1.pdf]

Genetics and Molecular Biology
https://doi.org/10.1590/1678-4685-GMB-2020-0173

Supplementary Material to "Differential expression of antioxidant system genes in honey bee (*Apis mellifera* L.) caste development mitigate ROS-mediated oxidative damage in queen larvae"

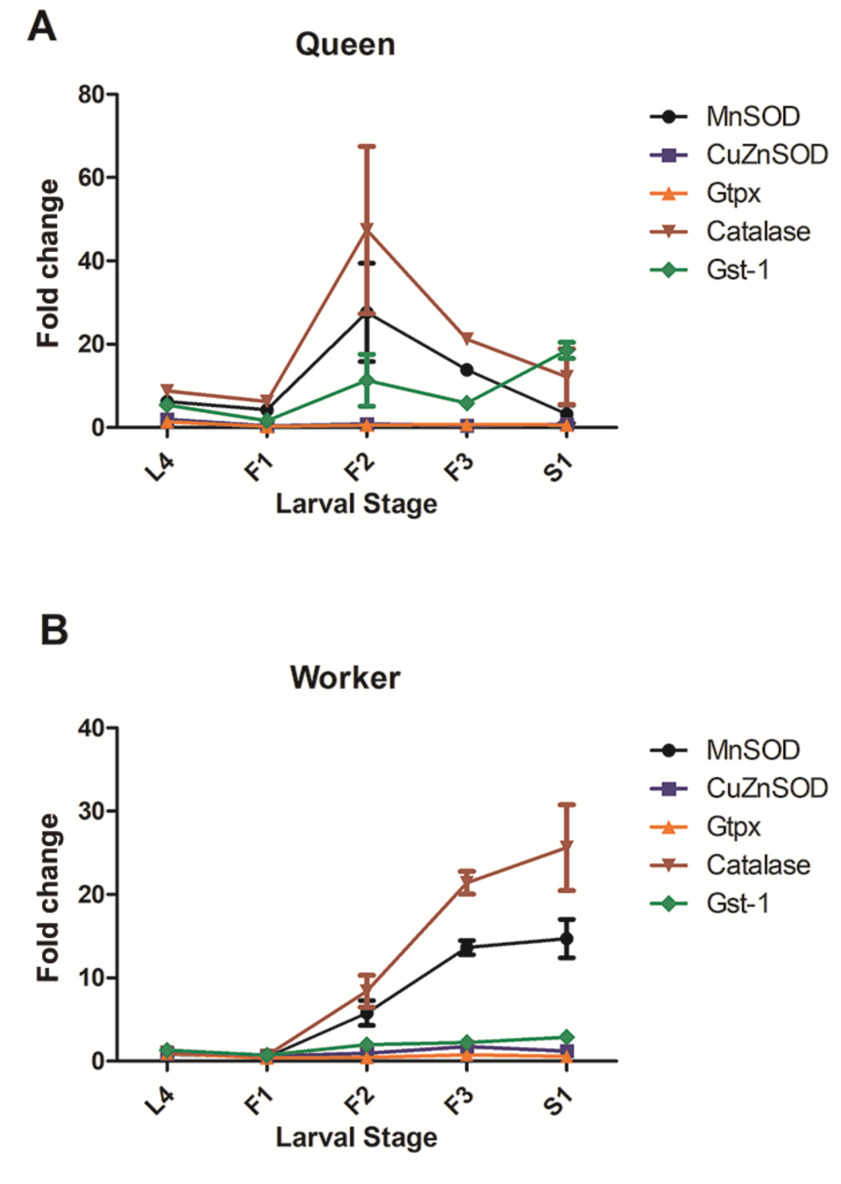

Figure S1 - Fold change of antioxidant genes *MnSOD*, *CuZnSOD*, *catalase*, *Gtpx* and *Gst-1* in *A. mellifera* queen (A) and worker (B) larvae. Shown are means and SEM of three biological samples per stage and caste, each analyzed in triplicate. Fold change was calculated by the $2^{-\Delta\Delta Ct}$ method (Livak and Schmittgen, 2001) using *rpl32* expression for normalization, and one of the fourth-instar worker samples as calibrator. The data are the same as those presented in Figure 3, but shown from a caste perspective.
